# 

**Published:** 1898-03-05

**Authors:** 


					THE HOSPITAL. ??The standai d ol highest purity."- Lancet. March 5th, 1&&S.
CADBURY'S ^ WJT Wm CADBURY'S
cocoa ? IJHI Jm cocoa
^ ABSOLUTELY PURE. ^ zL.; NO DRUQS OR ALKALIES USED.
'RHICH HOSPI TAL
No. 597. Vol. XXIII. [JES**] Saturday,
Mahcii 5tii, 1898.
[8DbB^0merm8,l P?ICB TWOPENCE.

				

